# Unraveling the Origin of Donor‐Like Effect in Bismuth–Telluride‐Based Thermoelectric Materials

**DOI:** 10.1002/smsc.202300082

**Published:** 2023-08-10

**Authors:** Feng Liu, Min Zhang, Pengfei Nan, Xin Zheng, Yuzheng Li, Kang Wu, Zhongkang Han, Binghui Ge, Xinbing Zhao, Chenguang Fu, Tiejun Zhu

**Affiliations:** ^1^ State Key Laboratory of Silicon and Advanced Semiconductor Materials School of Materials Science and Engineering Zhejiang University Hangzhou 310058 China; ^2^ Information Materials and Intelligent Sensing Laboratory of Anhui Province, Key Laboratory of Structure and Functional Regulation of Hybrid Materials of Ministry of Education, Institutes of Physical Science and Information Technology Anhui University Hefei 230601 China; ^3^ Shanxi-Zheda Institute of Advanced Materials and Chemical Engineering Taiyuan 030000 China

**Keywords:** bismuth-tellurides, donor-like effect, point defects, thermoelectric properties

## Abstract

The donor‐like effect, depicting the uncontrollable increase of electron density that can significantly alter the thermoelectric performance of both p‐type and n‐type polycrystalline Bi_2_Te_3_‐based materials, has long been an intriguing phenomenon, while its origin is still elusive. Herein, it is found that different from the common argument, the donor‐like effect in Bi_2_Te_3_‐based polycrystals is a result of the oxygen‐adsorption‐induced evolution of the point defects. The dominant point defect in stoichiometric zone‐melted Bi_2_Te_3_ ingot is the acceptor‐like $B{i^{\prime }}_{Te}$. During the fabrication of high‐strength polycrystals, the exposure of the powders to the air leads to their absorption of oxygen and the formation of secondary phase Bi_2_TeO_5_ in the following sintering process. This brings about a change of local chemical equilibrium and promotes the evolution of the intrinsic point defect from acceptor‐like $B{i^{\prime }}_{Te}$ to donor‐like $Te_{Bi}^{•}$. Notably, if the fabrication process is strictly controlled to minimize oxygen adsorption, the evolution of the point defects will be avoided, whereby the donor‐like effect disappears. Consequently, a reproducible high *zT* value of 1.0 at 325 K can be achieved in Bi_2_Te_2.7_Se_0.3_‐based polycrystals. These results highlight the importance of understanding the evolution of point defects, which is crucial for developing high‐performance Bi_2_Te_3_‐based polycrystals and corresponding fabrication processes.

## Graphical abstract

With a the defect calculation, the oxygen‐adsorption‐induced evolution of antisite defects from $B{i^{\prime }}_{Te}$ to $Te_{Bi}^{•}$ is argued to be responsible for the donor‐like effect in Bi_2_Te_3_‐based polycrystals. By strictly minimizing exposure to the air, a reproducible and high *zT* value of 1.0 can be obtained, which is beneficial for developing large‐scale fabrication processing with repeatable and good thermoelectric performance.

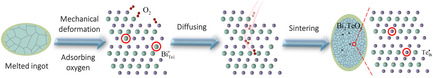

## 1 Introduction

Thermoelectric (TE) technology can realize mutual conversion between thermal energy and electricity, which has conventionally exhibited important applications in energy harvesting and solid‐state refrigeration.^[^ ^1^, ^2^, ^3^ ^]^ In recent years, the rapidly increasing demand for precise temperature control in various fields, including but not limited to optoelectronics, 5 G communications, and PCR thermal cyclers, further drives the development of TE materials that have high‐performance near‐room temperature. The conversion efficiency of a TE material is determined by the dimensionless figure of merit *zT*, $zT\;=S^{2}\sigma T/\kappa_{\text{tot}}$, where *S*, *σ*, *κ*_tot_, and *T* are the Seebeck coefficient, the electrical conductivity, the thermal conductivity (including the electronic contribution *κ*_e_ and the phonon contribution *κ*_L_), and the absolute temperature, respectively.

**Figure 1:**
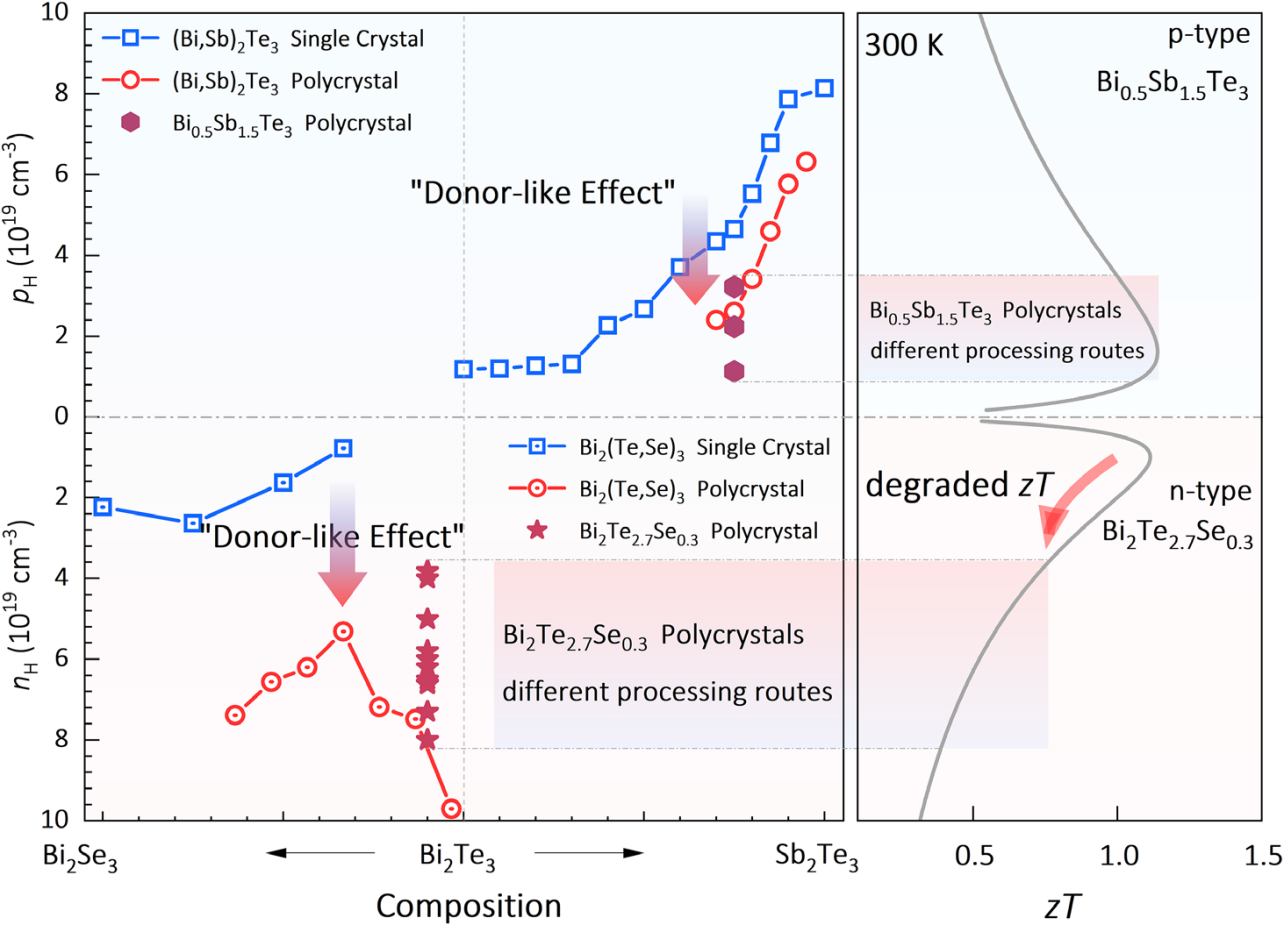
Carrier concentration versus composition for both single‐crystalline and polycrystalline Bi_2_(Te, Se)_3_ and (Bi, Sb)_2_Te_3_ alloys (left panel), and calculated carrier concentration dependence of *zT* for n‐type Bi_2_Te_2.7_Se_0.3_ and p‐type Bi_0.5_Sb_1.5_Te_3_ (right panel).^[^ ^6^, ^10^, ^12^, ^13^, ^14^, ^15^, ^16^, ^25^, ^26^ ^]^

Up to date, bismuth‐telluride (Bi_2_Te_3_)‐based alloys have been the only commercialized TE materials near room temperature.^[^ ^2^, ^4^ ^]^ The crystal structure (space group: $R\bar{3}m$) of Bi_2_Te_3_ features the periodically ordered quintuple‐layer (Te_1_–Bi–Te_2_–Bi–Te_1_) and van der Waals bonding between quintuple layers.^[^ ^5^ ^]^ In industry, the zone melting (ZM) method has long been utilized to produce both p‐type and n‐type Bi_2_Te_3_‐based TE alloys.^[^ ^6^ ^]^ Nevertheless, owing to the weak van der Waals bonding, cleavage and breakage of Bi_2_Te_3_‐based TE alloys are prone to occur during the assembly of TE modules, resulting in a lower yield rate and higher production cost.^[^ ^7^, ^8^ ^]^ This problem becomes even more serious when making TE micromodules that require rectangular TE legs down to a width of hundreds of micrometers. To improve machinability, powder metallurgy methods have gained much attention as a means to prepare high‐strength Bi_2_Te_3_‐based alloys utilizing the fine‐grain strengthening effect. However, the TE performance of the as‐sintered polycrystalline Bi_2_Te_3_‐based alloys might exhibit unexpectedly poor reproducibility due to the occurrence of the so‐called donor‐like effect, which depicts the uncontrollable increase of electron density of the as‐sintered alloys that had been subjected to the mechanical and thermal deformation processes.^[^ ^9^, ^10^ ^]^ The donor‐like effect can significantly induce a deviation from the optimal carrier concentration and deteriorate the *zT* of Bi_2_Te_3_‐based materials (**Figure** **1**).

As shown in the left panel of Figure 1, compared to the single crystals, the increased electron concentration is commonly observed for both (Bi, Sb)_2_Te_3_ and Bi_2_(Te, Se)_3_ polycrystals, which is conventionally thought to be the result of the donor‐like effect.^[^ ^11^ ^]^ For p‐type (Bi, Sb)_2_Te_3_ polycrystals (top panel of Figure 1), the donor‐like effect might help drop the over‐high hole concentration to the optimal range, beneficial for high *zT* values,^[^ ^12^ ^]^ whereas the donor‐like effect induces a triple to a quadruple increase in the electron concentration of n‐type Bi_2_(Te, Se)_3_ polycrystals (bottom panel of Figure 1), significantly deviating from the optimum value, leading to degraded *zT* values.^[^ ^10^ ^]^ It is worth mentioning that the donor‐like effect strongly depends on processing routes.^[^ ^13^ ^]^ As shown in Figure 1, the electron concentration of n‐type Bi_2_Te_2.7_Se_0.3_ polycrystals prepared by different methods varies from 3.8 × 10^19^ to 8 × 10^19^ cm^−3^, the hole concentration of p‐type Bi_0.5_Sb_1.5_Te_3_ polycrystals varies from 1.1 × 10^19^ to 3.2 × 10^19^ cm^−3^, indicating the poor reproducibility of TE performance in both p‐type and n‐type Bi_2_Te_3_‐based polycrystals.^[^ ^12^, ^13^, ^14^, ^15^, ^16^ ^]^ Hence, understanding the origin of the donor‐like effect is vital for finding an effective strategy that can control the carrier concentration and guarantee good reproducibility of the high TE performance for Bi_2_Te_3_‐based polycrystals.

It is worth noting that the increased electron concentration in polycrystalline Bi_2_Te_3_‐based polycrystalline alloys has been recognized for more than 60 years since the pioneering work by Schultz et al.^[^ ^17^ ^]^ Conventionally, the mechanical deformation‐induced evolution of point defects has been thought to be responsible for the donor‐like effect.^[^ ^11^, ^12^, ^13^, ^14^, ^17^, ^18^, ^19^, ^20^, ^21^, ^22^ ^]^ The dominant point defect in stoichiometric zone‐melted Bi_2_Te_3_‐based ingots is the acceptor‐like antisite defect $B{i^{\prime }}_{Te}$, making it a weak p‐type.^[^ ^23^ ^]^ When subjected to the mechanical deformation process, typically ball milling or hot deformation, the ingot will break through either the nonbasal or basal planes. The nonbasal plane gives on average 3 Te to 2 Bi vacancy‐interstitial pairs, while the basal plane slip gives only Te vacancy‐interstitial pairs, resulting in a ratio of Te interstitials or vacancies to Bi interstitials or vacancies greater than 3/2.^[^ ^17^ ^]^ In the subsequent sintering process, the existing $B{i^{\prime }}_{Te}$ is prone to occupying the Bi vacancy ${V^{\text{′′′}}}_{Bi}$, while the remaining Te vacancy $V_{Te}^{••}$ leads to the increased electron concentration in the as‐sintered polycrystals. Hence, the origin of the donor‐like effect was conventionally attributed to the mechanical deformation‐induced interaction of the point defects, which can be described by the following equation^[^ ^22^ ^]^

(1) $$2{V^{\text{′′′}}}_{Bi}+3V_{Te}^{••}+{{\text{Bi}}^{\prime }}_{Te}\to {V^{\text{′′′}}}_{Bi}+Bi_{Bi}^{\times }+4V_{Te}^{••}+6e^{\prime }$$

With this conventional awareness of the donor‐like effect, one can, to some extent, adjust the electron concentration of Bi_2_Te_3_‐based polycrystals by controlling the concentration of antisite defects or the strength of mechanical deformation,^[^ ^12^, ^13^, ^14^, ^24^ ^]^ for instance, the ball milling time.

Despite the success of this conventional explanation of the donor‐like effect, there are yet some elusive phenomena, both experimentally and theoretically, that cannot be well clarified. First, due to the smaller differences of the electronegativity *χ* and the covalent radius *r* between Sb and Te, alloying Sb into (Bi, Sb)_2_Te_3_ should decrease the formation energy of antisite defects $B{i^{\prime }}_{Te}$/ $S{b^{\prime }}_{Te}$,^[^ ^12^, ^18^ ^]^ whereas alloying Se into Bi_2_(Te, Se)_3_ will result in the increased formation energy of antisite defects.^[^ ^14^ ^]^ Thus, according to Equation (1), one should expect a larger increase in the electron concentration of p‐type (Bi, Sb)_2_Te_3_ polycrystals compared to the n‐type Bi_2_(Te, Se)_3_ polycrystals because the former has a higher concentration of antisite defects which should facilitate the occurrence of the donor‐like effect. However, the experimental results show that n‐type polycrystals exhibit a larger increase of electron concentration (Figure 1).^[^ ^12^, ^25^, ^26^ ^]^ Second, the defect calculation results suggest that antisite defects ($B{i^{\prime }}_{Te}$ and $Te_{Bi}^{•}$) are the dominant native point defects in Bi_2_Te_3_ while the formation energy of vacancies (${V^{\text{′′′}}}_{Bi}$ and $V_{Te}^{••}$) is significantly higher.^[^ ^27^, ^28^, ^29^ ^]^ If the initial chemical environment is Bi‐rich, the formation energy of $B{i^{\prime }}_{Te}$ is the lowest, making Bi_2_Te_3_ p‐type, while $Te_{Bi}^{•}$ is dominant if Te is rich, making Bi_2_Te_3_ n‐type. These conclusions are in line with the experimental results of Bi_2_Te_3_ single crystals.^[^ ^23^, ^25^, ^26^ ^]^ According to Equation (1), $V_{Te}^{••}$ is supposed to be the dominant point defect in deformed Bi_2_Te_3_ polycrystal, which however, cannot find convincing theoretical support from the defect calculations. Very recently, Tao et al. reported that the donor‐like effect is strongly related to both the vacancy defects (${V^{\text{′′′}}}_{Bi}$ and $V_{Te}^{••}$) induced by the fracturing process and oxygen in the air, and they argued that oxygen can promote the mechanical deformation‐induced donor‐like effect, with the interaction described by the equation^[^ ^30^ ^]^

(2) $$2{V^{\text{′′′}}}_{Bi}+3V_{Te}^{••}+2B{i^{\prime }}_{Te}+O_{2}\to 2Bi_{Bi}^{\times }+2O_{Te}^{\times }+3V_{Te}^{••}+8e^{\prime }$$

According to Equation (2), $V_{Te}^{••}$ is still the origin of the increased electron concentration in Bi_2_Te_3_‐based polycrystals where oxygen promotes the happening of this process. This also conflicts with the defect calculations suggesting that antisite defects ($B{i^{\prime }}_{Te}$ and $Te_{Bi}^{•}$) have the lowest formation energy. In short, these results manifest that the origin of the deformation‐induced donor‐like effect is still elusive, both experimentally and computationally.^[^ ^12^, ^13^, ^14^, ^17^, ^18^, ^25^, ^26^, ^27^, ^28^, ^29^ ^]^ This motivates us to carry out further studies to understand this intriguing donor‐like effect in Bi_2_Te_3_‐based polycrystals.

In this work, the increase of electron concentration, i.e., the donor‐like effect, is observed as long as the powders are exposed to air during the preparation process. Together with the transport measurements, transmission electron microscopy analysis, and defect calculations, it is found that the increased electron concentration relates to the oxygen absorption‐induced secondary phase, resulting in the evolution of the dominant antisite defects from the acceptor‐like $B{i^{\prime }}_{Te}$ to the donor‐like $Te_{Bi}^{•}$ in the matrix. Importantly, by reducing the oxygen adsorption to eliminate the donor‐like effect, a reproducible high *zT* value of 1.0 at 325 K is obtained in n‐type Bi_2_Te_2.7_Se_0.3_. These findings pave the way for the controllable large‐scale fabrication of Bi_2_Te_3_‐based polycrystals with both good TE and mechanical properties.

## 2 Results and Discussion

### 2.1 The Correlation Between the Donor‐Like Effect and the Air Exposure

**Figure 2:**
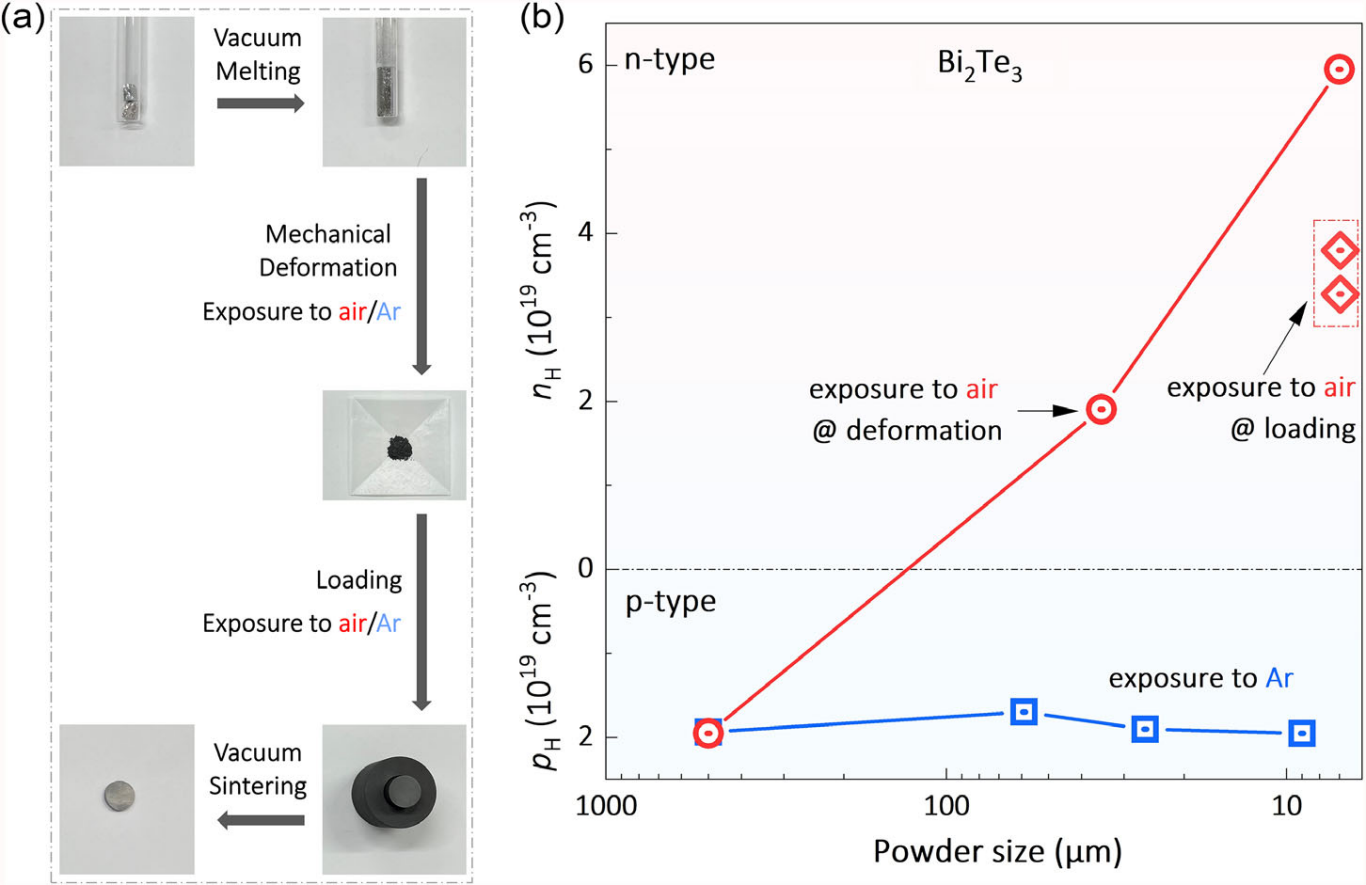
a) Typical preparation process of Bi_2_Te_3_‐based polycrystals. b) Powder size dependence of *n*_H_ for Bi_2_Te_3_ polycrystals prepared with exposure to air/Ar in different processing steps.

A typical process for fabricating Bi_2_Te_3_‐based polycrystal is shown in **Figure** **2**a. High‐purity raw elements are mixed homogeneously and placed into a quartz tube in which the Bi_2_Te_3_‐based ingot is obtained after vacuum melting. Then, fine‐grain powders are prepared using the as‐melted ingots by mechanical deformation, typically ball milling, which are subsequently loaded into a graphite die and compacted by hot pressing (HP) or spark plasma sintering. During the whole preparation process, mechanical deformation has been conventionally thought to induce the donor‐like effect,^[^ ^22^ ^]^ as mentioned above (Equation (1)). It has also been reported that the oxygen can cause the change in the carrier concentration of Bi_2_Te_3_‐based polycrystals, but the mechanism is elusive.^[^ ^17^, ^31^, ^32^ ^]^ Very recently, Tao et al. reported that air/oxygen plays a crucial role in inducing the donor‐like effect (Equation (2)), in addition to mechanical deformation.^[^ ^30^ ^]^ The nitrogen was also proven to be independent of the donor‐like effect.^[^ ^30^ ^]^ Based on these existing results in the literature, two processes, i.e., both mechanical deformation and exposure to air/oxygen, are found to be related to the donor‐like effect. Here, to give a glimpse into the possible origin of the donor‐like effect, the Bi_2_Te_3_‐based polycrystals with enhanced mechanical deformation, i.e., decreased grain sizes, were first prepared under both Ar and air atmosphere, respectively, for comparative analysis.

In the first set of experiments, Bi_2_Te_3_ polycrystals were prepared with the powders exposed to air during the ball milling process, i.e., the powders were loaded into the ball‐milling jars in the ambient air. As shown in Figure 2b, the p‐to‐n transition occurs and the electron concentration increases with enhanced mechanical deformations, i.e., decreased grain sizes, indicating that the donor‐like effect happens. This phenomenon is in line with many previous results when fabricating n‐type Bi_2_Te_3_‐based polycrystals,^[^ ^11^, ^22^, ^33^ ^]^ suggesting that mechanical deformation might contribute to the donor‐like effect. Then, to figure out the role of the atmosphere, a second set of experiments were carried out: another batch of Bi_2_Te_3_‐polycrystals was then prepared with the powders exposed to high‐purity Ar during mechanical deformation, while the other processes were strictly controlled to avoid exposure to the air. Surprisingly, it can be seen that the conduction type keeps p‐type and the hole concentration is nearly unchanged even if undergoing severe mechanical deformation, i.e., the average grain size drops from 500 to 9 μm. This demonstrates that the donor‐like effect disappears when the preparation process is strictly controlled to avoid exposure to the air. It is worth noting that this phenomenon was also observed by Tao et al.^[^ ^30^ ^]^ These results together highlight the vital role of air exposure of the powders in the occurrence of the donor‐like effect.

To further examine the impact of air exposure, we carried out a third set of experiments: the fine‐grain powders were ball‐milled under high‐purity Ar while they were only exposed to the ambient air for about 5 min before hot‐pressing. As shown in Figure 2b, although only a short‐time exposure to the air, the donor‐like effect is still observable: the conduction type becomes n‐type while the electron concentration reaches about 4 × 10^19^ cm^−3^. This result suggests that there is a high correlation between the donor‐like effect and the exposure of the powders to the ambient air, even for only several minutes. During the conventional preparation of Bi_2_Te_3_ polycrystals, particularly large‐scale industrial production, the unconscious exposure of the powders to the ambient air and thus the occurrence of the donor‐like effect seems to be unavoidable, which explains why the TE performance of the as‐sintered polycrystalline Bi_2_Te_3_‐based alloys exhibit unexpectedly poor reproducibility.

### 2.2 The Existence of the Bi_2_TeO_5_ Secondary Phase

**Figure 3:**
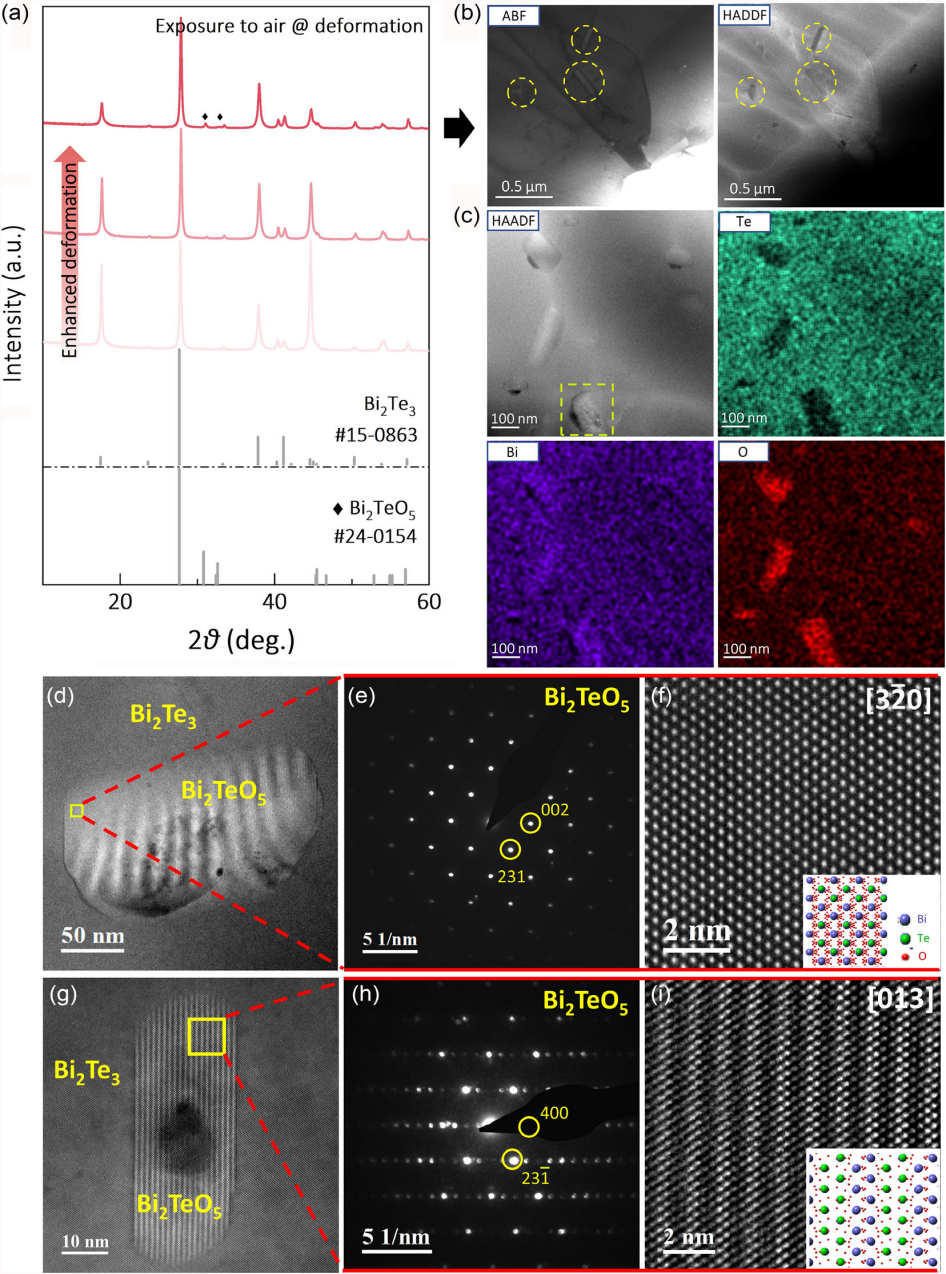
Microstructural analysis for the samples prepared under exposure to the air. a) The XRD patterns. b) TEM analysis on the sample that experienced the strongest mechanical deformation: low‐magnification ABF and HAADF images. c) EDS mapping, containing Bi, Te, and O. d) Enlarged HAADF image of the secondary phase in (c). e,f) Corresponding nanobeam diffraction pattern and atomic‐scale HAADF image. g–i) Enlarged HAADF image, nanobeam diffraction pattern, and atomic‐scale HAADF image of the secondary phase in another region.

To investigate how the donor‐like effect correlates to air exposure, the chemical composition of the studied polycrystals was first analyzed by the electron probe microanalyzer (EPMA). As shown in Table S1, Supporting Information, there is no obvious composition fluctuation in the matrix that relates to the donor‐like effect within the detection limit. Further, the X‐ray diffraction (XRD) analysis was utilized to examine the phase structure. For the samples prepared under Ar atmosphere (the second set of experiments), all samples crystallize in a rhombohedral phase (R$\bar{3}$ m) and no secondary phase is observed (Figure S1, Supporting Information). In contrast, for the samples prepared under exposure to the air (the first set of experiments), two diffraction peaks that cannot be indexed to Bi_2_Te_3_ gradually become visible in the samples with enhanced mechanical deformation (**Figure** **3**a). With further analysis, this second phase is preliminarily identified to Bi_2_TeO_5_.

To further examine the composition and structure of the secondary phase, the transmission electron microscopy (TEM) analysis was carried out on the air‐exposure sample that experienced the strongest mechanical deformation. By the contrast between annular bright‐field (ABF) and high‐angle annular dark‐field (HAADF) images in Figure 3b, the existence of the secondary phase is confirmed, consistent with the XRD result (Figure 3a). The energy‐dispersive X‐ray spectroscopy (EDS) mappings in Figure 3c further show that the secondary phase is enriched in Bi and O, but deficient in Te, compared to the Bi_2_Te_3_ matrix. To examine the phase structure, nanobeam diffraction was performed on the secondary phase. The diffraction pattern of the secondary phase in Figure 3d is the same as the simulated one of Bi_2_TeO_5_ (space group: Abm2) (Figure 3e). Moreover, the crystal structure model of Bi_2_TeO_5_ matches well with the atomic‐scale HAADF image (Figure 3f). Notably, the secondary phase of Bi_2_TeO_5_ can be observed in different zones (Figure 3g–i and S2, Supporting Information), supporting that the Bi_2_TeO_5_ phase exists in samples that experienced the exposure to air during the preparation process.

In comparison, the TEM images of the sample prepared with only exposure to Ar show no obvious secondary phase and the diffraction pattern is the same as the simulated one of Bi_2_Te_3_ (Figure S3, Supporting Information). Hence, the major difference in the microstructure of Bi_2_Te_3_ polycrystals prepared in air/Ar is the oxygen‐induced Bi_2_TeO_5_ phase, which may be correlated with the occurrence of the donor‐like effect (Figure 2b).

### 2.3 The Oxygen‐Induced Evolution of Antisite Defects

The oxygen‐induced Bi_2_TeO_5_ phase is an insulator with a bandgap of 2.53 eV,^[^ ^34^ ^]^ suggesting that it cannot directly provide additional electrons to cause the donor‐like effect. Nevertheless, the generation of the Bi_2_TeO_5_ phase can lead to local composition fluctuation. The formation of intrinsic point defects in Bi_2_Te_3_ might be subsequently influenced by the altered chemical environment, resulting in increased electron concentration.

**Figure 4:**
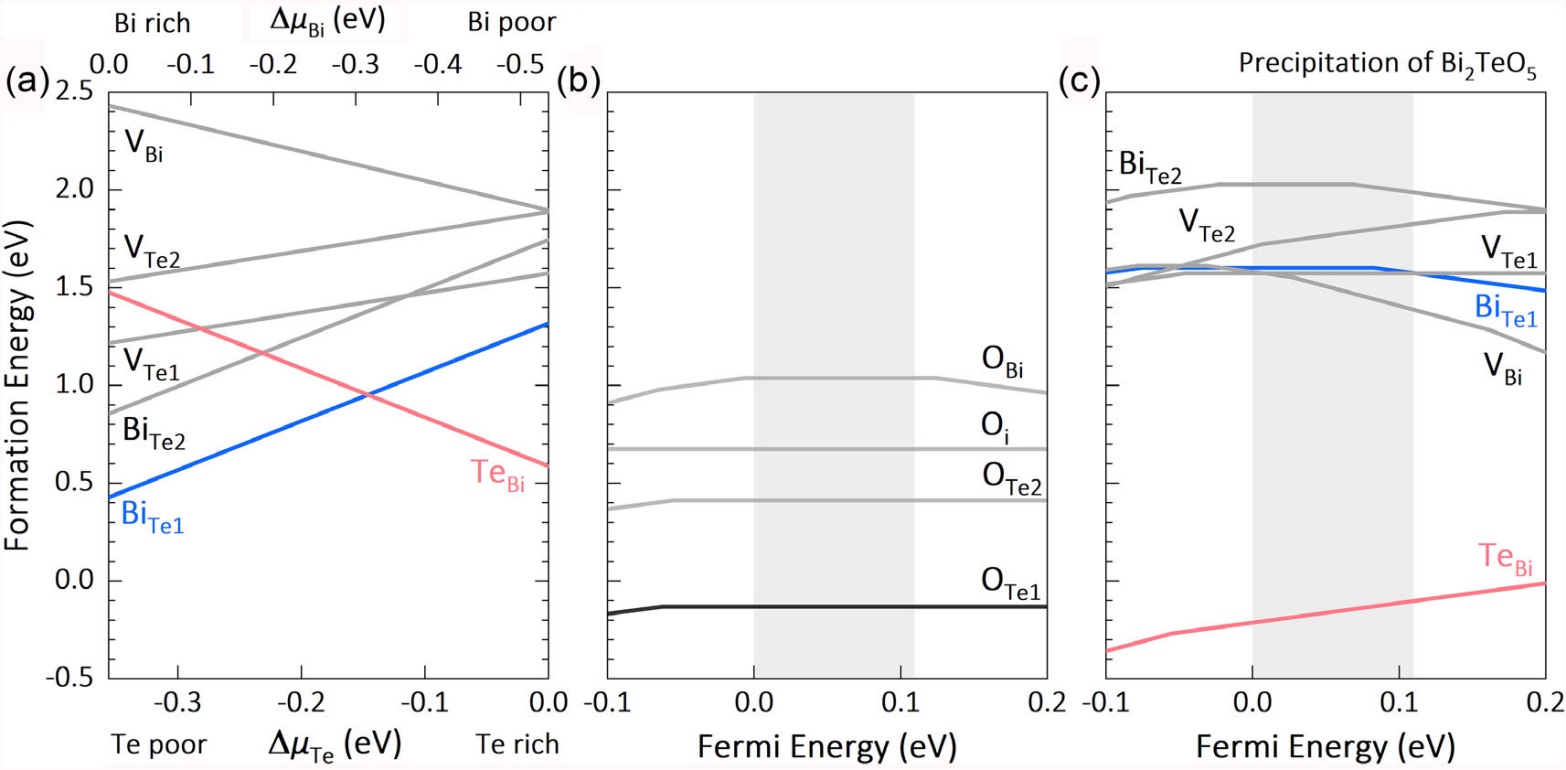
a) Formation energy for the possible native point defects in Bi_2_Te_3_ as a function of the relative chemical potentials of Te (bottom axis) and Bi (upper axis). b) Formation energy for possible substitutional or interstitial sites for O in Bi_2_Te_3_. c) Formation energy for the possible native point defects in Bi_2_Te_3_, under the existence of Bi_2_TeO_5_. The shaded areas in (b) and (c) represent the bandgap of Bi_2_Te_3_.

To examine the oxygen‐induced evolution of point defects in Bi_2_Te_3_, the defect calculations based on density functional theory (DFT) were employed to obtain the formation energy of point defects in different chemical environments. Here, the considered intrinsic point defects in Bi_2_Te_3_ contain both vacancies and antisite defects.^[^ ^27^ ^]^ First, the point defects formation energy Δ*H* under the Bi‐rich or Te‐rich environment is displayed in **Figure** **4**a. It can be seen that the Δ*H* of the acceptor‐like antisite defect $B{i^{\prime }}_{Te1}$ is the lowest in the Bi‐rich chemical environment, and the donor‐like antisite defect $Te_{Bi}^{•}$ is dominant in the Te‐rich chemical environment, which is in line with the previous report by Adham et al.^[^ ^27^ ^]^ The calculation results suggest that the conduction behavior of Bi_2_Te_3_ can be converted from p‐type to n‐type by increasing Te atomic percentage, i.e., changing the chemical environment from the Bi‐rich to Te‐rich, as also supported by the experimental results of Bi_2_Te_3_‐based single crystals.^[^ ^19^, ^23^ ^]^ These results indicate that the carrier type and carrier concentration of Bi_2_Te_3_‐based crystals is very sensitive to the chemical environment of the matrix.

Then, the oxygen‐induced change of the chemical environment is considered in the defect calculations. The boundary conditions for defining the chemical potentials of Bi, Te, and O are discussed in Supporting Information. The site preference of O in Bi_2_Te_3_ is first studied (Figure 4b), including substitutional and interstitial sites (Figure S4b, Supporting Information). It is shown that the substitution of O at the Te site is highly preferred. The replacement finally leads to the formation of the Bi_2_TeO_5_ phase in the Bi_2_Te_3_ matrix.

Considering that the Bi_2_Te_3_ polycrystals prepared in Ar are p‐type (Figure 2b), the chemical environment should be Bi‐rich. When exposed to air during preparation, the EDS mappings (Figure 3c) show that the Bi_2_TeO_5_ phase is rich in O and Bi, deficient in Te, compared to the Bi_2_Te_3_ matrix, suggesting that the chemical composition of the matrix surrounding the Bi_2_TeO_5_ phase should be rich in Te, but deficient in O and Bi. The generation of the Bi_2_TeO_5_ phase leads to a local chemical environment that is Te‐rich. Consequently, it can be seen that the Δ*H* of $Te_{Bi}^{•}$ is dramatically decreased, and the Δ*H* of $B{i^{\prime }}_{Te1}$ is greatly increased (Figure 4c). The dominant antisite defects change from $B{i^{\prime }}_{Te}$ to $Te_{Bi}^{•}$. The occurrence of $Te_{Bi}^{•}$ results in increased electron concentration, i.e., the occurrence of the donor‐like effect.

**Figure 5:**
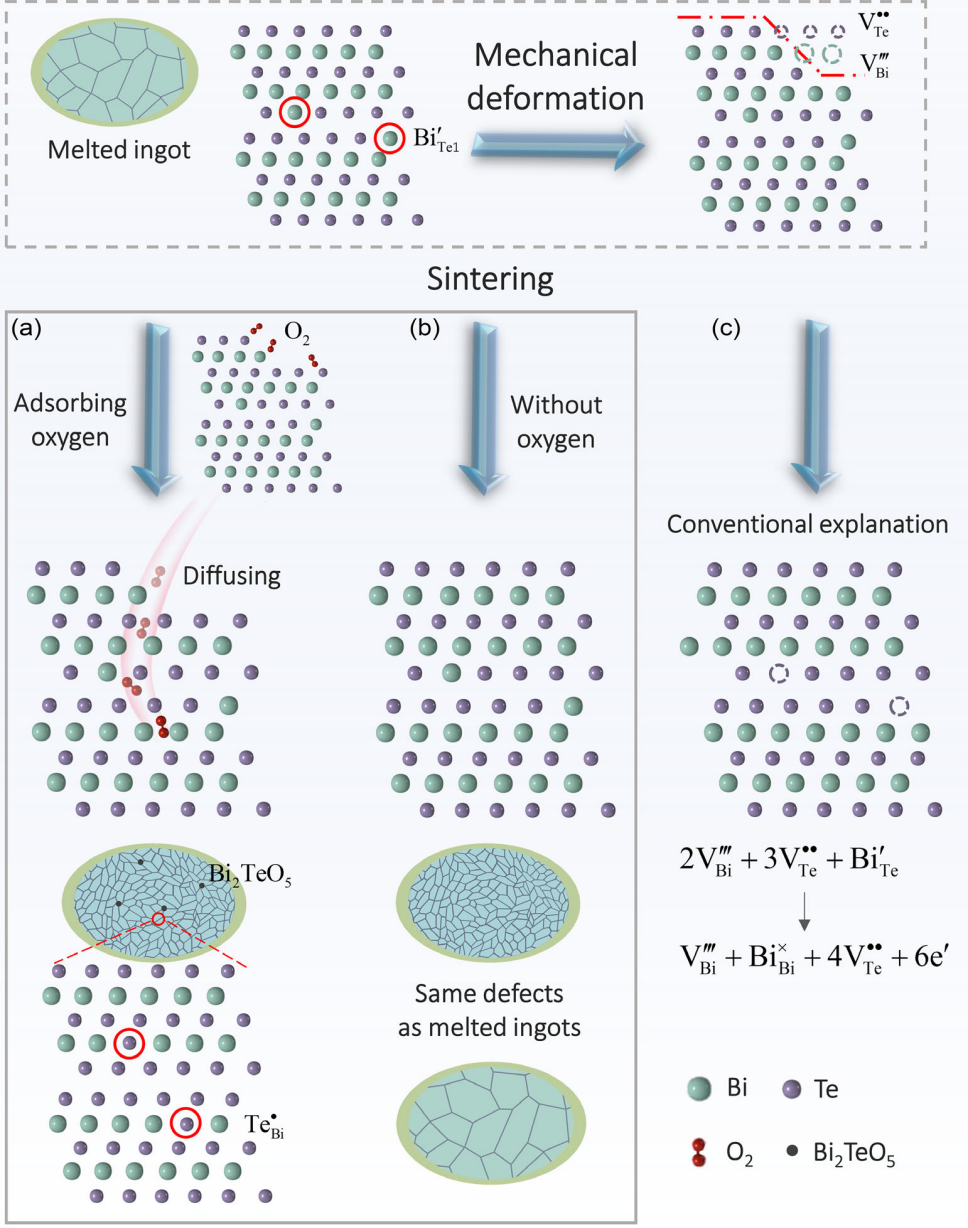
Schematic diagram of the evolution of point defects with (a) or without (b) exposure to oxygen in preparation processes. c) The conventional explanation of the donor‐like effect.

Based on the above discussions, the oxygen‐induced evolution of the dominant point defects in Bi_2_Te_3_ polycrystals is proposed (**Figure** **5**a). First, the as‐melted Bi_2_Te_3_ ingot is p‐type due to the dominant point defect of $B{i^{\prime }}_{Te}$. The mechanical deformation processes cause basal plane slip and nonbasal plane slip. Nonbasal plane slip results in off‐cut atomic terraces containing vacancies of Bi and Te (the top panel of Figure 5).^[^ ^17^, ^35^ ^]^ Moreover, previous research proposed that the vacancies are active sites for adsorbing oxygen.^[^ ^17^ ^]^ In high‐temperature sintering, the adsorbed oxygen can diffuse into Bi_2_Te_3_ through basal planes, and preferably interact with Bi because of the stronger Bi–O bonding compared to Te–O bonding.^[^ ^35^ ^]^ Then, the generation of the Bi_2_TeO_5_ phase changes the local chemical environment of the matrix from Bi‐rich to Te‐rich. Under such a circumstance, the donor‐like antisite defect $Te_{Bi}^{•}$ might occur owing to the dramatically decreased formation energy. Finally, the increased concentration of $Te_{Bi}^{•}$ causes the occurrence of the donor‐like effect. Further, the oxygen‐induced donor‐like effect is more significant for samples that experienced enhanced deformation and exposure to the air (Figure 2b), which is because of that there are more active sites for adsorbing oxygen. Once oxygen adsorption is avoided, as shown in Figure 5b, the vacancies induced by deformation can be eliminated due to the recovery effect during high‐temperature sintering.^[^ ^12^, ^17^ ^]^ The chemical environment is the same as the as‐melted ingot. Hence, the hole concentration stays unchanged (Figure 2b). It is worth noting that some previous works reported that the addition of interstitial copper and boron could help inhibit the donor‐like effect.^[^ ^36^, ^37^ ^]^ According to our point of view, this might be because of the reduced active sites for oxygen adsorption owing to the existence of interstitial cooper and boron.

Conventionally, the donor‐like effect is attributed to mechanical deformation induced $V_{Te}^{••}$ (Figure 5c). However, as displayed in Figure 4a,c, and S5, Supporting Information, the formation energy of $V_{Te}^{••}$ in different chemical environments is always significantly higher than the antisite defects (both $B{i^{\prime }}_{Te}$ and $Te_{Bi}^{•}$). Hence, there is no convincing theoretical support from the point defect calculations to prove that $V_{Te}^{••}$ is the dominant point defect in the thermodynamically stable Bi_2_Te_3_ polycrystals. In short, we argue that the oxygen adsorption‐induced evolution of antisite defects might be the real origin of the donor‐like effect. If oxygen adsorption is avoided, the hole concentration of polycrystals will stay unchanged. The so‐called “donor‐like effect” is extrinsic and avoidable. Understanding the origin of the donor‐like effect will help to realize reproducibly superior TE performance in Bi_2_Te_3_‐based polycrystals.

### 2.4 Reproducible High TE Performance

In this section, the influence of oxygen‐induced donor‐like effect on the TE performance of Bi_2_Te_3_‐based polycrystals is systematically investigated. Samples are prepared with exposure to the air and Ar, respectively. The Bi_2_Te_2.7_Se_0.3_ composition is chosen because of its favorable band structure, bandgap, and relatively low lattice thermal conductivity *κ*_L_.^[^ ^38^ ^]^ The TeI_4_ is chosen as the n‐type dopant to modulate electron concentration.^[^ ^39^ ^]^

**Figure 6:**
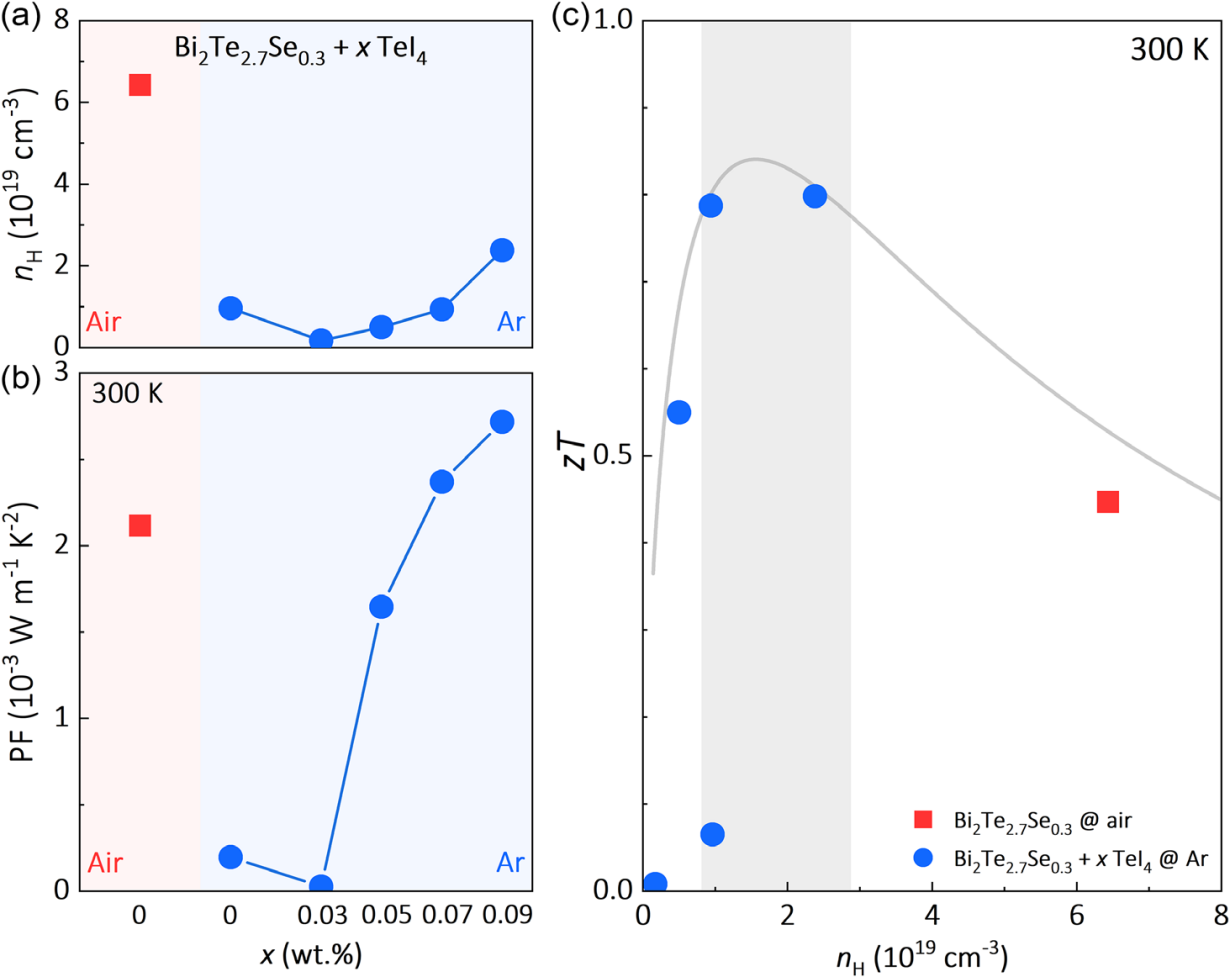
a) *n*_H_, b) PF, and c) *zT* for Bi_2_Te_2.7_Se_0.3_ + *x* wt% TeI_4_ samples prepared in air and Ar. The line in (c) is fitted by TE properties of Bi_2_Te_2.7_Se_0.3_ + 0.09 wt% TeI_4_ sample.

In the case of exposure to air, an oxygen‐induced donor‐like effect in the Bi_2_Te_2.7_Se_0.3_ polycrystal can increase the electron concentration by six times compared to that prepared in Ar (**Figure** **6**a), which leads to the increased electrical conductivity (Figure S6, Supporting Information) and power factor PF (Figure 6b). But the over‐high electron concentration severely deviates from the optimal range shown in Figure 6c. Eventually, the *zT* value only reaches 0.44 owing to the high electronic thermal conductivity and the unoptimized power factor (Figure S6, Supporting Information). In air, the oxygen adsorption is uncontrollable during the preparation processes, resulting in the poor reproducibility of the TE performance of n‐type Bi_2_Te_3_‐based polycrystals (Figure 1).

For Bi_2_Te_3_ polycrystals prepared in Ar, it has been shown that the carrier concentration is independent of preparation processes (Figure 2b). Further, their *S* values show good reproducibility (Figure S7, Supporting Information), manifesting that avoiding oxygen adsorption is imperative for obtaining reproducible TE performance. Therefore, another batch of Bi_2_Te_2.7_Se_0.3_ + *x* wt% TeI_4_ polycrystals were prepared in Ar to ensure reproducibility. With increased TeI_4_ dopant content, the electron concentration first decreases and then increases (Figure 6a). Under low doping content of TeI_4_, anion vacancies might be compensated by Te, leading to decreased electron concentration. When *x* > 0.03, the dominant point defects change to $I_{\text{Te}/\text{Se}}^{•}$, causing increased electron concentration. Their PF shows a similar trend (Figure 6b). When *x* = 0.07 and 0.09, their electron concentration lies in the optimal range and the *zT* values at 300 K greatly improve to 0.8 (Figure 6c).

**Figure 7:**
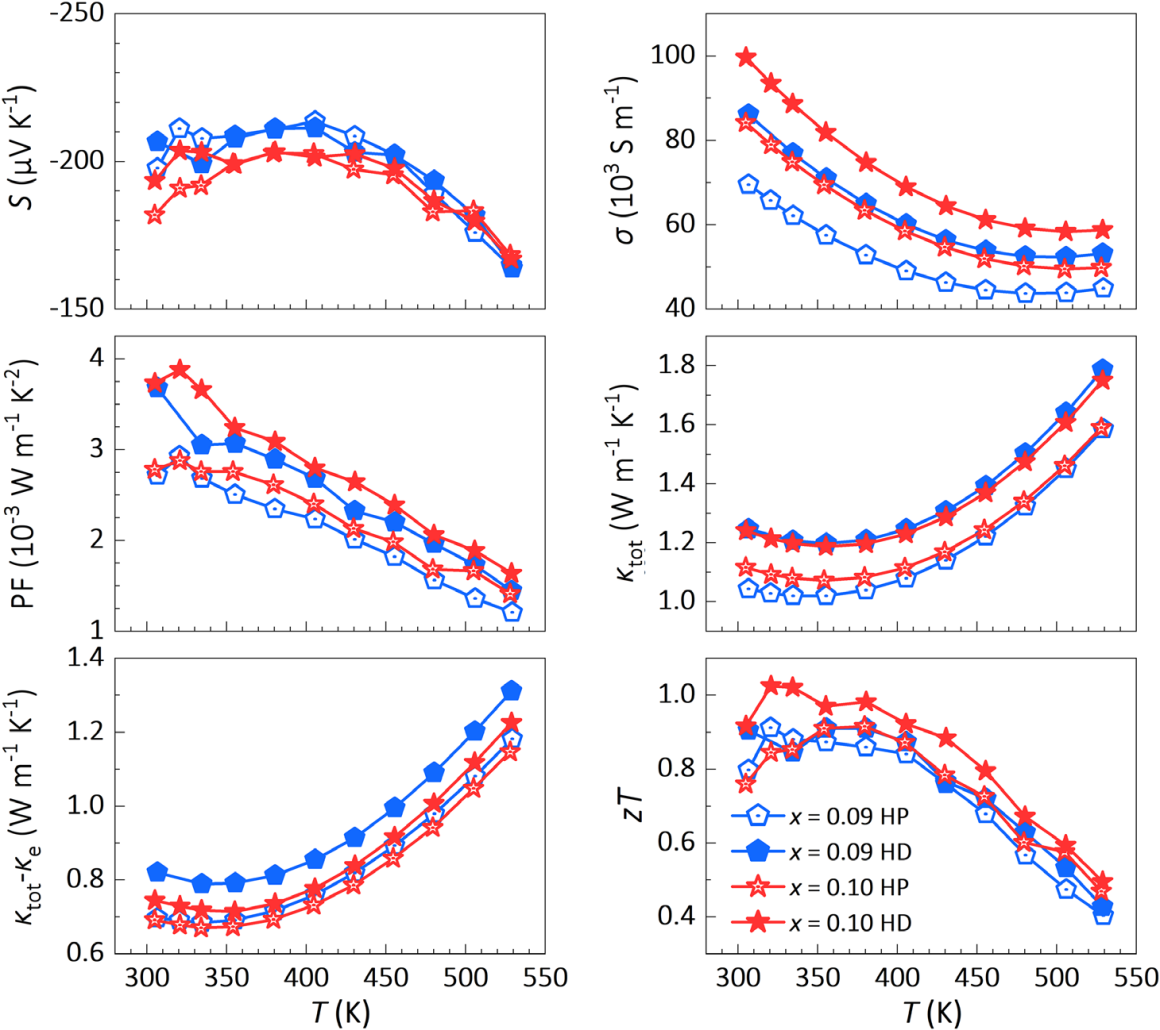
Temperature dependences of *S*, *σ*, PF, *κ*_tot_, *κ*_tot_ − *κ*_e,_ and *zT* for HP and HD Bi_2_Te_2.7_Se_0.3_ + 0.09 and 0.1 wt% TeI_4_ polycrystals prepared in Ar.

To further enhance PF and *zT*, the hot deformation (HD) process was conducted for sample *x* = 0.09.^[^ ^21^ ^]^ It was reported that the electron concentration increases after the hot deformation.^[^ ^9^ ^]^ Here, the electron concentration remains almost unchanged after HD because the oxygen‐induced donor‐like effect is avoided. Another hot‐deform experiment was carried out on sample *x* = 0.10 sample, showing the same result. Their electron concentration constantly lies in the optimum range and the carrier mobility is enhanced after HD due to the enhanced texture (**Table** **1**), beneficial for improving *zT* values (**Figure** **7**). Finally, the reproducible and high *zT* values of 0.9 at 300 K and 1.0 at 325 K for n‐type Bi_2_Te_2.7_Se_0.3_ polycrystal with 0.10 wt% TeI_4_ are obtained.

**Table 1:** The *n*_H_ and *μ*_H_ for HP and HD *x* = 0.09, 0.10 samples

	HP		HD	
	*n*_H_ [10^19^ cm^3^]	*μ*_H_ [cm^2^ V^−1^ s^−1^]	*n*_H_ [10^19^ cm^3^]	*μ*_H_ [cm^2^ V^−1^ s^−1^]
*x* = 0.09	2.38	184	2.29	235
*x* = 0.10	2.98	176	2.77	209

## 3 Conclusion

The origin of the donor‐like effect in Bi_2_Te_3_‐based polycrystals is explored and clarified. The occurrence of the donor‐like effect is very sensitive to the exposure of the powders to the ambient air. It is found that the oxygen adsorption of the powders induces the generation of the Bi_2_TeO_5_ phase in the following sintering process, resulting in the composition fluctuation and the changed local chemical environment of the matrix from Bi‐rich to Te‐rich. With the defect calculation, the oxygen adsorption‐induced evolution of antisite defects from $B{i^{\prime }}_{Te}$ to $Te_{Bi}^{•}$ is argued to be responsible for the donor‐like effect. Importantly, the donor‐like effect is extrinsic and can be avoided by strictly minimizing exposure to the air. Finally, by eliminating the oxygen‐induced donor‐like effect, a repeatable and high *zT* value of 1.0 at 325 K is obtained. This work unravels the origin of the donor‐like effect that is crucial for developing large‐scale fabrication processing of Bi_2_Te_3_‐based polycrystals with repeatable and good TE performance.

## 4 Experimental Section

4.1

4.1.1

##### Sample Synthesis

Highly pure element chunks of Bi, Te, Se (5 N, Emei Semiconductor Materials Research Institute), and TeI_4_ powders (metal 99%, Alfa Aesar China) were weighted according to the stoichiometric Bi_2_Te_3_ and Bi_2_Te_2.7_Se_0.3_ + *x* wt% TeI_4_ (*x* = 0, 0.03, 0.05, 0.07, 0.09, 0.1). Then the mixtures were sealed into clean quartz tubes below 10^−3^ Pa and melted in the Muffle furnace at 1273 K for 12 h. The tubes were rocked twice to ensure composition homogeneity during melting and finally cooled in the air to obtain ingots. The zone‐melted Bi_2_Te_3_ ingots were also fabricated for comparison and the experimental details were described elsewhere.^[^ ^6^ ^]^

##### For Bi_2_Te_3_ Ingots

A bar with dimensions of 2.5 × 2.5 × 11 mm^3^ was cut from ingots for TE properties measurement. Ingots were crushed into pieces and divided into three individual parts. One was directly loaded into Φ 20 mm die and vacuum hot‐pressed at 773 K for 30 min with 80 MPa uniaxial pressure (4505 J, MRF). Another was hand grounded and sieved through 100 mesh and 300 mesh sieves, respectively. The last was ball‐milled into fine powders by planetary ball milling at 400 rpm for 3 h. The powders preparation processes were conducted in the Ar and air, respectively. Finally, the three obtained powders were hot‐pressed into dense bulks. Apart from that, the ball‐milled powders in the Ar atmosphere were loaded into a die in the air atmosphere and then hot pressed.

##### For Bi_2_Te_2.7_Se_0.3_ + *x* wt% TeI_4_ Ingots

Ingots were ball‐milled into powders and hot‐pressed into Φ 20 mm bulks. Hot deformation processes are as follows. The powders were first sintered into Φ 12.6 mm bulks and subsequently deformed to Φ 15, and Φ 20 mm. The above processes were all carried out in the Ar atmosphere or under vacuum to avoid exposure to air/oxygen.

##### Structure Characterization

The phase structures were investigated by XRD on an Aeris DY866 diffractometer with Cu k*α* radiation. The distribution of the powder size was measured on a Beckman Coulter LS‐230 apparatus. The elemental composition was analyzed by the EPMA (JEOL JXA‐8100) using a wavelength dispersive spectroscope. TEM images, EDS mapping, and ABF were carried out using a JEOL F200 microscope operated at 200 kV. The atomic resolution HAADF image was obtained using a FEI Titan Themis Z microscope equipped with probe and image correctors operated at 300 kV. Samples for TEM characterization were prepared by traditional mechanical polishing, dimpling, and argon ion milling with a liquid nitrogen stage.

##### TE Properties Measurements

The electrical conductivity *σ* and Seebeck coefficient *S* were simultaneously measured on the commercial Linseis LSR‐3 system. The thermal conductivity *κ* was calculated using *κ* = *ρDC*_p_, where *ρ* is the density of the sample determined by the Archimedes method, *C*_p_ is the specific heat estimated by Dulong–Petit law, and *D* is the thermal diffusivity measured by the Netzsch LFA 467 instrument. The Hall coefficient *R*_H_ from 10 to 300 K was collected on a Mini Cryogen Free Measurement System (Cryogenic Limited, UK) with the magnetic field varied between ±4.0 T. The Hall carrier concentration *n*_H_ and Hall mobility *μ*_H_ were determined via *n*_H_ = 1/*eR*_H_ and *μ*_H_ = *σR*_H_, respectively. Notably, all TE properties were measured perpendicular to the hot press direction.^[^ ^40^ ^]^

##### DFT Calculations

To obtain the point defect formation energy Δ*H*, the DFT calculations were employed using the Vienna ab initio Simulation Package with the projector augmented‐wave method. The local density approximation (LDA) was suggested as the exchange‐correlation functional.^[^ ^41^ ^]^ A 2 × 2 × 1 supercell for a unit cell of Bi_6_Te_9_ (24 Bi atoms and 36 Te atoms) was used in the self‐consistent calculations.^[^ ^27^ ^]^ A plane‐wave energy cutoff of 400 eV and an energy convergence criterion of 10^−4^ eV for self‐consistency were adopted. All of the atomic positions were relaxed to equilibrium until the calculated Hellmann−Feynman force on each atom was less than 10^−2^ eV Å^−1^. The Monkhorst–Pack uniform k‐point sampling with *k* = 120/*L* (*L* is the corresponding lattice parameter) was used in self‐consistent static calculations. The Δ*H* was calculated by the following equations

(3) $$\Delta H=E_{\text{tot}}^{\text{def}}-E_{\text{tot}}^{p}-\sum_{i}n_{i}\mu_{i}+q(E_{F}+E_{V})$$

where $E_{\text{tot}}^{\text{def}}$ and $E_{\text{tot}}^{p}$ are the energy of the defective crystal and perfect crystal, respectively. The *n*_*i*_ is the change in the number of atoms of species *i*, i.e., of either Bi or Te, in the unit cell relative to the ideal bulk one. The *n*_*i*_ > 0 indicates adding atoms to the supercell. The *μ*_*i*_ is the chemical potential of the element *i*. The last term gives the variation in the energy due to the charge state *q* of a defect.

In this work, an assumption is made, whereby the system is in equilibrium with a bulk Bi_2_Te_3_ reservoir.^[^ ^29^ ^]^ Hence, the chemical potentials of Bi and Te are related through

(4) $$E^{\text{bulk}}(Bi_{2}Te_{3})=2\mu_{Bi}+3\mu_{Te}$$

or

(5) $$\Delta H_{f}(Bi_{2}Te_{3})=3\Delta \mu_{Te}+2\Delta \mu_{Bi}$$

where $E^{\text{bulk}}(Bi_{2}Te_{3})$ is the energy of one formula unit of Bi_2_Te_3_ and Δ*μ*_*i*_ is given with respect to the value of a solid phase *μ*_*i*_^0^, i.e., the absolute value of the chemical potential *μ*_*i*_ = Δ*μ*_*i*_ + *μ*_*i*_^0^. The Δ*H*_*f*_ is defined by

(6) $$\Delta H_{f}=E_{\text{tot}}^{\text{bulk}}-\sum_{i}\mu_{i}^{0}$$

## Conflict of Interest

The authors declare no conflict of interest.

## Supporting information

Supplementary Material

## Data Availability

The data that support the findings of this study are available from the corresponding author upon reasonable request.

## References

[1] J. He , T. M. Tritt , Science 2017, 357, eaak9997. 28963228 10.1126/science.aak9997

[2] J. P. Heremans , R. J. Cava , N. Samarth , Nat. Rev. Mater. 2017, 2, 17049.

[3] F. Wang , J. D. Harindintwali , Z. Yuan , M. Wang , F. Wang , S. Li , Z. Yin , L. Huang , Y. Fu , L. Li , S. X. Chang , L. Zhang , J. Rinklebe , Z. Yuan , Q. Zhu , L. Xiang , D. C. W. Tsang , L. Xu , X. Jiang , J. Liu , N. Wei , M. Kastner , Y. Zou , Y. S. Ok , J. Shen , D. Peng , W. Zhang , D. Barcelo , Y. Zhou , Z. Bai , et al., Innovation 2021, 2, 100180. 34877561 10.1016/j.xinn.2021.100180 PMC8633420

[4] X. F. Tang , Z. W. Li , W. Liu , Q. J. Zhang , C. Uher , Interdiscip. Mater. 2022, 1, 88.

[5] J. R. Drabble , C. H. L. Goodman , J. Phys. Chem. Solids. 1958, 5, 142.

[6] R. S. Zhai , Y. H. Wu , T. J. Zhu , X. B. Zhao , Cryst. Growth Des. 2018, 18, 4646.

[7] Y. Zheng , H. Y. Xie , S. C. Shu , Y. G. Yan , H. Li , X. F. Tang , J. Electron. Mater. 2013, 43, 2017.

[8] M. Haras , T. Skotnicki , Nano Energy 2018, 54, 461.

[9] L. P. Hu , Y. Zhang , H. J. Wu , Y. M. Liu , J. Q. Li , J. He , W. Q. Ao , F. S. Liu , S. J. Pennycook , X. R. Zeng , Adv. Funct. Mater. 2018, 28, 1803617.

[10] Q. Zhang , T. Fang , F. Liu , A. R. Li , Y. H. Wu , T. J. Zhu , X. B. Zhao , Chem. Asian J. 2020, 15, 2775. 32696486 10.1002/asia.202000793

[11] T. S. Oh , D. B. Hyun , N. V. Kolomoets , Scripta Mater. 2000, 42, 849.

[12] L. P. Hu , T. J. Zhu , X. H. Liu , X. B. Zhao , Adv. Funct. Mater. 2014, 24, 5211.

[13] Q. Zhang , B. C. Gu , Y. H. Wu , T. J. Zhu , T. Fang , Y. X. Yang , J. D. Liu , B. J. Ye , X. B. Zhao , ACS Appl. Mater. Interfaces 2019, 11, 41424. 31612710 10.1021/acsami.9b15198

[14] Y. Pan , T. R. Wei , C. F. Wu , J. F. Li , J. Mater. Chem. C. 2015, 3, 10583.

[15] Y. H. Wu , R. S. Zhai , T. J. Zhu , X. B. Zhao , Mater. Today Phys. 2017, 2, 62.

[16] Y. H. Wu , Y. Yu , Q. Zhang , T. J. Zhu , R. S. Zhai , X. B. Zhao , Adv. Sci. 2019, 6, 1901702. 10.1002/advs.201901702 PMC6839625 31728293

[17] J. M. Schultz , J. P. McHugh , W. A. Tiller , J. Appl. Phys. 1962, 33, 2443.

[18] T. J. Zhu , L. P. Hu , X. B. Zhao , J. He , Adv. Sci. 2016, 3, 1600004. 10.1002/advs.201600004 PMC5071658 27818905

[19] G. R. Miller , C. Y. Li , J. Phys. Chem. Solids 1965, 26, 173.

[20] R. Ionescu , J. Jaklovszky , N. Nistor , A. Chiculita , Phys. Stat. Sol. A 1975, 27, 27.

[21] L. P. Hu , X. H. Liu , H. H. Xie , J. J. Shen , T. J. Zhu , X. B. Zhao , Acta Mater. 2012, 60, 4431.

[22] J. Navratil , Z. Stary , T. Plechacek , Mater. Res. Bull. 1996, 31, 1559.

[23] C. B. Satterthwaite , R. W. Ure , Phys. Rev. 1957, 108, 1164.

[24] F. Liu , Y. H. Wu , Q. Zhang , T. J. Zhu , X. B. Zhao , Rare Metals 2020, 40, 513.

[25] V. U. Birkholz , Z. Naturforschung A 1958, 13, 780.

[26] Z. Stary , J. Horak , M. Stordeur , M. Stolzer , J. Phys. Chem. Solids 1988, 49, 29.

[27] A. Hashibon , C. Elsässer , Phys. Rev. B. 2011, 84, 144117.

[28] D. West , Y. Y. Sun , H. Wang , J. Bang , S. B. Zhang , Phys. Rev. B. 2012, 86, 121201.

[29] J. M. Zhang , W. M. Ming , Z. G. Huang , G. B. Liu , X. F. Kou , Y. B. Fan , K. L. Wang , Y. G. Yao , Phys. Rev. B. 2013, 88, 235131.

[30] Q. Tao , H. Wu , W. Pan , Z. Zhang , Y. Tang , Y. Wu , R. Fan , Z. Chen , J. Wu , X. Su , X. Tang , ACS Appl. Mater. Interfaces 2021, 13, 60216. 34874703 10.1021/acsami.1c19357

[31] J. K. Lee , J. H. Son , S. D. Park , S. Park , M. W. Oh , Mater. Lett. 2018, 230, 211.

[32] S. K. Li , M. H. Chu , W. M. Zhu , R. Wang , Q. Wang , F. S. Liu , M. Gu , Y. G. Xiao , F. Pan , Nanoscale. 2020, 12, 1580. 31859305 10.1039/c9nr07591g

[33] J. H. Qiu , Y. G. Yan , T. T. Luo , K. C. Tang , L. Yao , J. Zhang , M. Zhang , X. L. Su , G. J. Tan , H. Y. Xie , M. G. Kanatzidis , C. Uher , X. F. Tang , Energy Environ. Sci. 2019, 12, 3106.

[34] B. Chen , X. Z. Wang , J. Q. Li , Q. H. Xiong , C. H. Zhang , J. Mater. Chem. C. 2018, 6, 10435.

[35] D. Music , K. Chang , P. Schmidt , F. N. Braun , M. Heller , S. Hermsen , P. J. Pollmann , T. Schulzendorff , C. Wagner , J. Phys.: Condens. Matter. 2017, 29, 485705. 29120869 10.1088/1361-648X/aa945f

[36] W. S. Liu , Q. Y. Zhang , Y. C. Lan , S. Chen , X. Yan , Q. Zhang , H. Wang , D. Z. Wang , G. Chen , Z. F. Ren , Adv. Energy Mater. 2011, 1, 577.

[37] C. H. Zhang , X. J. Geng , B. Chen , J. Q. Li , A. Meledin , L. P. Hu , F. S. Liu , J. G. Shi , J. Mayer , M. Wutting , O. Cojocaru-Miredin , Y. Yu , Small. 2021, 17, e2104067. 34541782 10.1002/smll.202104067

[38] C. H. Champness , W. B. Muir , P. T. Chiang , Can. J. Phys. 1967, 45, 3611.

[39] S. Y. Wang , G. J. Tan , W. J. Xie , G. Zheng , H. Li , J. H. Yang , X. F. Tang , J. Mater. Chem. 2012, 22, 20943.

[40] W. J. Xie , J. He , S. Zhu , T. Holgate , S. Y. Wang , X. F. Tang , Q. J. Zhang , T. M. Tritt , J. Mater. Res. 2011, 26, 1791.

[41] T. Fang , X. Li , C. L. Hu , Q. Zhang , J. Yang , W. Q. Zhang , X. B. Zhao , D. J. Singh , T. J. Zhu , Adv. Funct. Mater. 2019, 29, 1900677.

