# Supplementary material for: Unraveling the Origin of Donor‐Like Effect in Bismuth–Telluride‐Based Thermoelectric Materials
Source: Small Sci. 2023 Aug 10;5(3):2300082. doi: 10.1002/smsc.202300082 (PMC12245077; doi:10.1002/smsc.202300082)
Supplement: Supplementary file 1 — Supplementary Material [file SMSC-5-2300082-s001.pdf]

Supporting Information

Unraveling the origin of donor-like effect in bismuth-telluride based thermoelectric materials

Feng Liu, Min Zhang, Pengfei Nan, Xin Zheng, Yuzheng Li, Kang Wu, Zhongkang Han, Binghui Ge, Xinbing Zhao, Chenguang Fu*, Tiejun Zhu*

F. Liu, M. Zhang, X. Zheng, Y. Z. Li, Prof. Z. K. Han, Prof. X. B. Zhao, Prof. C. G. Fu, Prof. T. J. Zhu

State Key Laboratory of Silicon and Advanced Semiconductor Materials, School of Materials Science and Engineering, Zhejiang University, Hangzhou 310058, China

E-mail: chenguang_fu@zju.edu.cn; zhutj@zju.edu.cn

Dr. P. F. Nan, K. Wu, Prof. B. H. Ge

Information Materials and Intelligent Sensing Laboratory of Anhui Province, Key Laboratory of Structure and Functional Regulation of Hybrid Materials of Ministry of Education, Institutes of Physical Science and Information Technology, Anhui University, Hefei 230601, China

Prof. T. J. Zhu

Shanxi-Zheda Institute of Advanced Materials and Chemical Engineering, Taiyuan 030000, China

Table S1 The actual composition of the matrix from the EPMA analysis. S, σ, n_H at 300 K for polycrystals prepared with exposure to air/Ar during the ball milling process.

	Samples	Actual composition	S (μV/K)	n_H (10^{19} cm^{-3})	σ (10^3 S/m)
**Exposure to air**	Bi_2Te_3-7 μm	Bi_2Te_3	-107.03	5.95(n)	195.64
	Bi_2Te_3-35 μm	$Bi_{2.01}Te_{2.99}$	-151.26	1.91(n)	81.49
	Bi_2Te_3-500 μm	$Bi_{2.01}Te_{2.99}$	196.23	2.53(p)	69.05
**Exposure to Ar**	Bi_2Te_3-9 μm	Bi_2Te_3	196.01	1.95(p)	20.17
	Bi_2Te_3-26 μm	$Bi_{1.99}Te_{3.01}$	209.42	1.90(p)	46.26
	Bi_2Te_3-59 μm	$Bi_{2.01}Te_{2.99}$	196.55	1.70(p)	49.17
	Bi_2Te_3-500 μm	$Bi_{2.01}Te_{2.99}$	208.74	1.94(p)	61.71
	Bi_2Te_3-M	$Bi_{2.01}Te_{2.99}$	208.42	/	45.20
	Bi_2Te_3-ZM	/	200.80	/	98.54

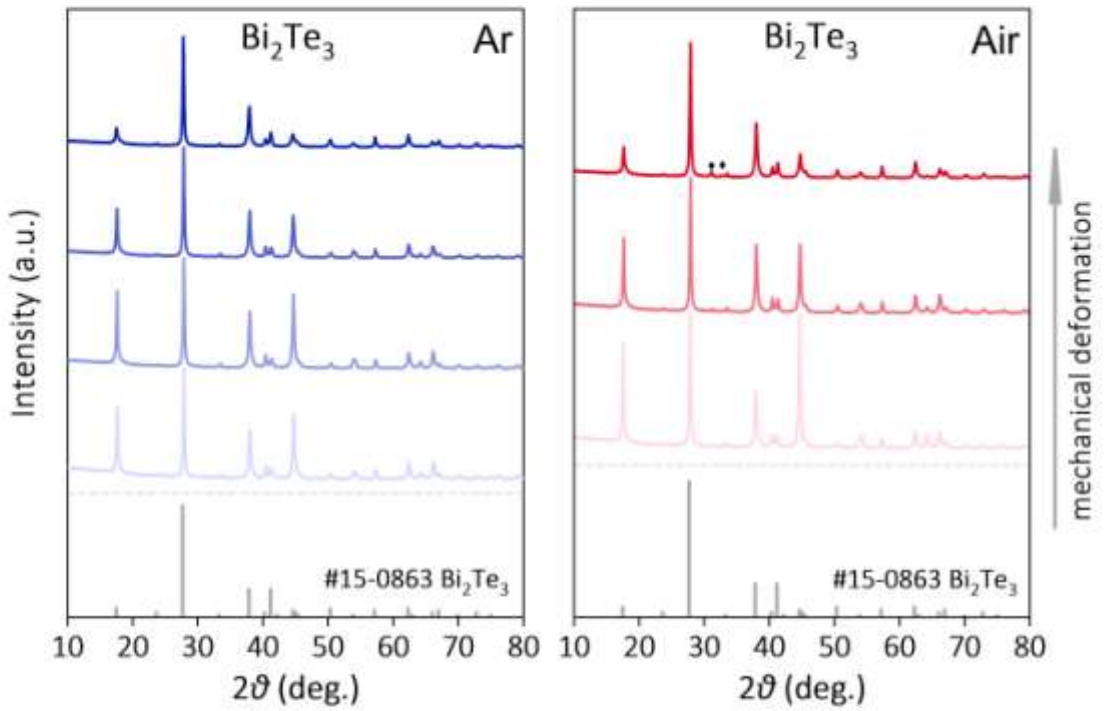

Fig. S1 The XRD patterns of Bi_2Te_3 bulk samples which were prepared in Ar and air, respectively.

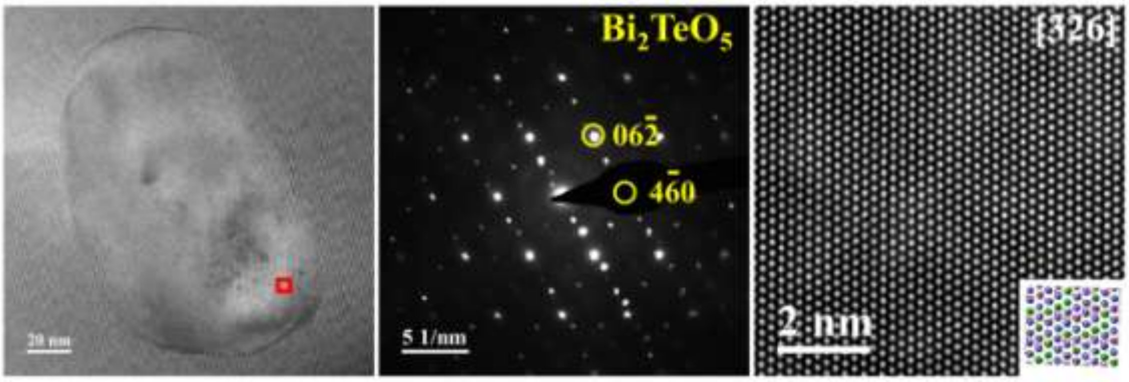

Fig. S2 The enlarged HAADF-STEM image, nano beam diffraction pattern and atomic-scale HAADF image for Bi_2TeO_5 in another zone.

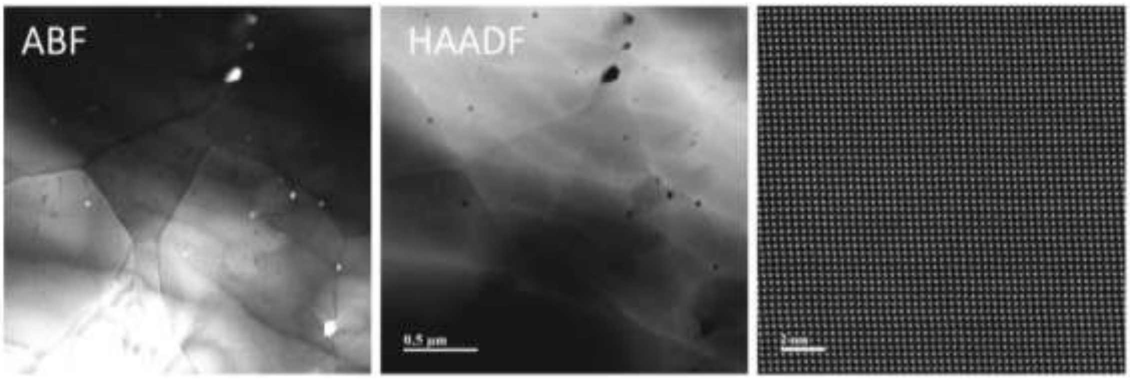

Fig. S3 The STEM images of samples prepared under Ar atmosphere.

The definement of chemical potential under precipitation of Bi_2TeO_5

The adopted boundary conditions are as follows:

$$\Delta\mu_{\mathrm{Bi}}, \Delta\mu_{\mathrm{Te}}, \Delta\mu_{\mathrm{O}} \le 0$$
$$2\Delta\mu_{\mathrm{Bi}} + \Delta\mu_{\mathrm{Te}} + 5\Delta\mu_{\mathrm{O}} \ge \Delta H_f(\mathrm{Bi_2TeO_5})$$
$$2\Delta\mu_{\mathrm{Bi}} + 3\Delta\mu_{\mathrm{O}} \le \Delta H_f(\mathrm{Bi_2O_3})$$
$$\Delta\mu_{\mathrm{Te}} + 2\Delta\mu_{\mathrm{O}} \le \Delta H_f(\mathrm{TeO_2})$$
$$\Delta\mu_{\mathrm{Te}} + 2\Delta\mu_{\mathrm{O}} \le \Delta H_f(\mathrm{TeO_3})$$
$$2\Delta\mu_{\mathrm{Bi}} + 3\Delta\mu_{\mathrm{Te}} \le \Delta H_f(\mathrm{Bi_2Te_3})$$

These equations aim to ensure that Bi_2TeO_5 is the main phase, and other competing phases, such as Bi_2Te_3, Bi_2O_3, TeO_2 and TeO_3, cannot precipitate.[1] The shaded areas in Fig.S5a represent the optional range for $\Delta\mu_{\mathrm{Bi}}$ and $\Delta\mu_{\mathrm{Te}}$, and $\Delta\mu_{\mathrm{O}}$, which are defined by $2\Delta\mu_{\mathrm{Bi}} + \Delta\mu_{\mathrm{Te}} + 5\Delta\mu_{\mathrm{O}} = \Delta H_f(\mathrm{Bi_2TeO_5})$. According to XRD, EPMA and TEM results, A, B, and C points are chosen to calculate the ΔH. At these points,

the chemical environments are Te-rich, but relatively deficient for Bi and O. The results for A point are shown in **Figs. 4b** and **4c**. The results for B and C points are shown in Fig. S5.

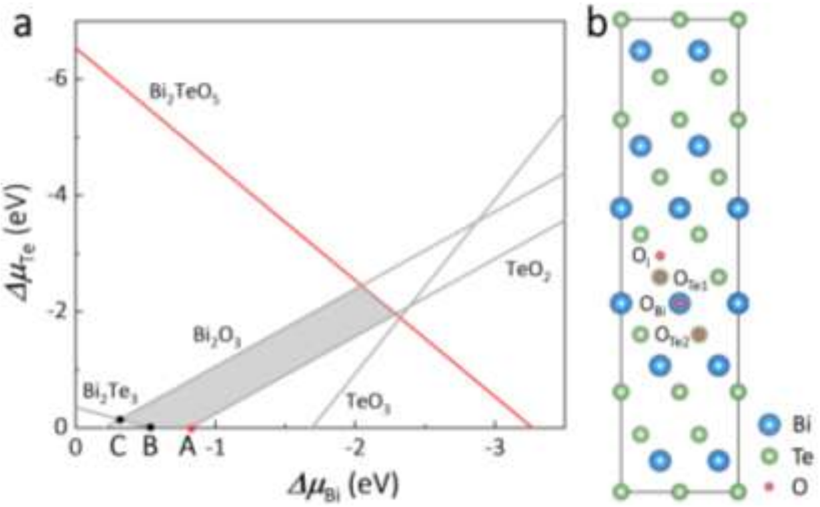

Fig. S4 **(a)** The optional range for $\Delta\mu_{Bi}$ and $\Delta\mu_{Te}$. **(b)** The possible substitutional or interstitial sites for O in Bi_2Te_3.

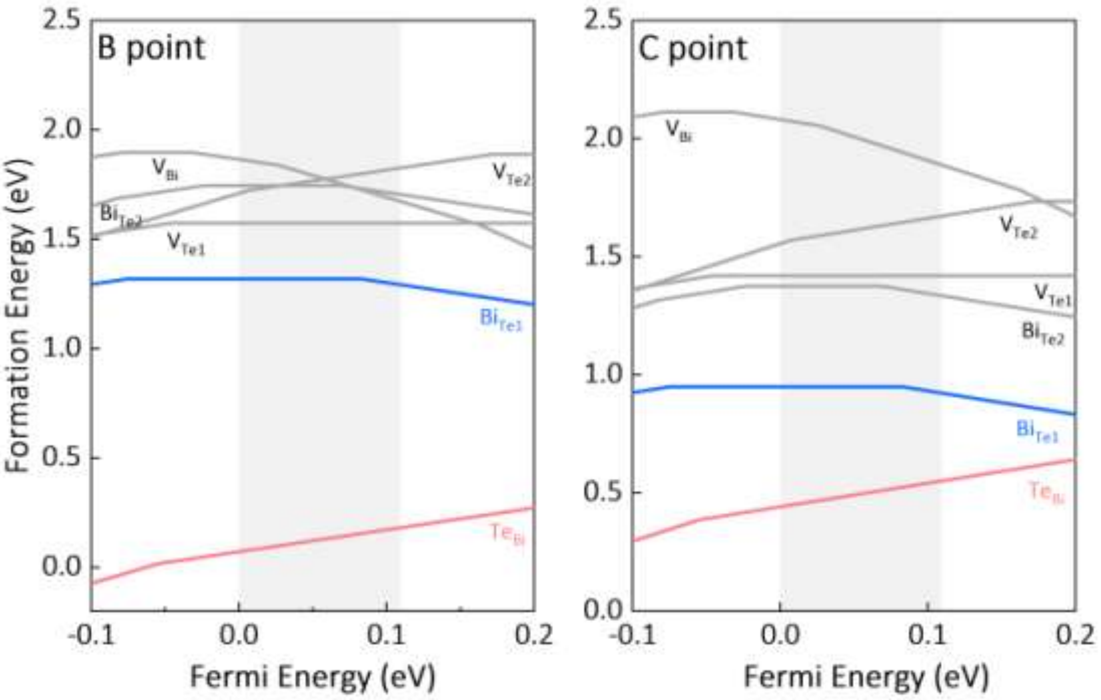

Fig. S5 The formation energies for the intrinsic point defects in Bi_2Te_3, at B and C points.

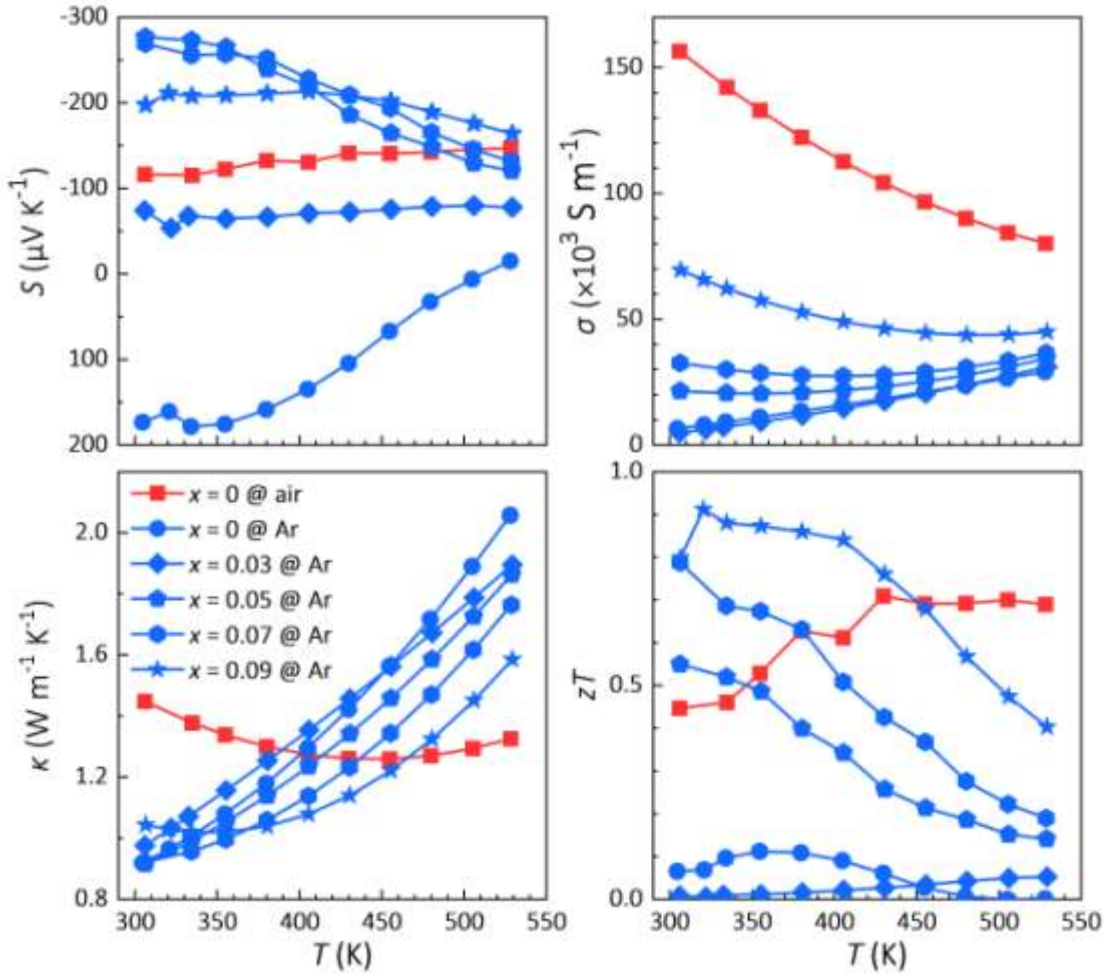

Fig. S6 Thermoelectric performance for $Bi_2Te_{2.7}Se_{0.3}$ + x wt.% TeI_4 polycrystals prepared in Ar and air.

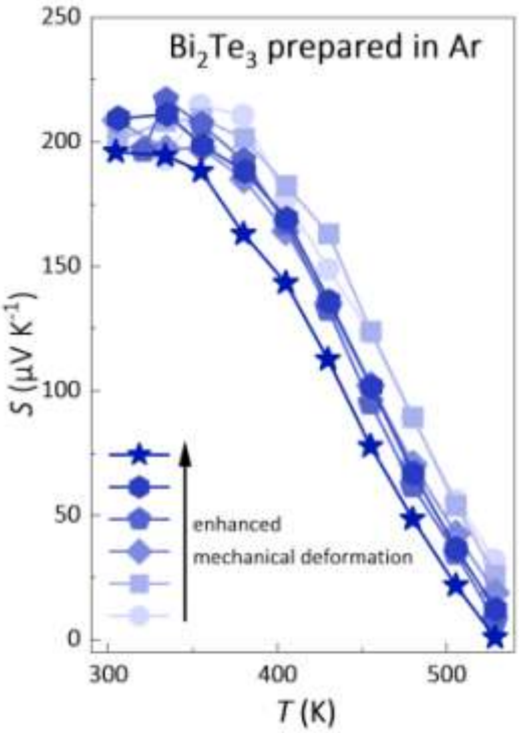

Fig. S7 Temperature dependence of *S* for Bi_2Te_3 polycrystals prepared in Ar with enhanced mechanical deformation.

References

[1] J. M. Zhang, W. M. Ming, Z. G. Huang, G. B. Liu, X. F. Kou, Y. B. Fan, K. L. Wang, Y. G. Yao. Stability, electronic, and magnetic properties of the magnetically doped topological insulators Bi_2Se_3, Bi_2Te_3, and Sb_2Te_3. Phys. Rev. B. 2013, 88(23):235131.
